# Sr-Doping All-Inorganic CsPbBr_3_ Perovskite Thick Film for Self-Powered X-ray Detectors

**DOI:** 10.3390/ma16051783

**Published:** 2023-02-21

**Authors:** Chuanqi Liu, Wen Zhang, Dingyu Yang, Haibo Tian, Jun Zhu

**Affiliations:** 1 College of Physics, Sichuan University, Chengdu 610065, China; 2 College of Optoelectronic Engineering, Chengdu University of Information Technology, Chengdu 610225, China

**Keywords:** X-ray, self-powered detectors, perovskite thick film, Sr-doped CsPbBr_3_

## Abstract

The all-inorganic perovskite cesium lead bromine (CsPbBr_3_) has attracted much attention in the field of X-ray detectors because of its high X-ray absorption coefficient, high carrier collection efficiency, and easy solution preparation. The low-cost anti-solvent method is the main method to prepare CsPbBr_3_; during this process, solvent volatilization will bring a large number of holes to the film, leading to the increase of defects. Based on the heteroatomic doping strategy, we propose that Pb^2+^ should be partially replaced by Sr^2+^ to prepare leadless all-inorganic perovskite. The introduction of Sr^2+^ promoted the ordered growth of CsPbBr_3_ in the vertical direction, increased the density and uniformity of the thick film, and achieved the goal of CsPbBr_3_ thick film repairing. In addition, the prepared CsPbBr_3_ and CsPbBr_3_:Sr X-ray detectors were self-powered without external bias, maintaining a stable response during on and off states at different X-ray dose rates. Furthermore, the detector base on 160 µm CsPbBr_3_:Sr had a sensitivity of 517.02 µC Gy_air_^−1^ cm^−3^ at zero bias under the dose rate of 0.955 µGy ms^−1^ and it obtained a fast response speed of 0.053–0.148 s. Our work provides a new opportunity to produce cost-effective and highly efficient self-powered perovskite X-ray detectors in a sustainable way.

## 1. Introduction

X-ray is widely used in medical, military, security, and material testing and other fields [1,2], due to its high energy and strong penetration ability; therefore, the detection of X-ray dose rate is particularly important. Materials for X-ray detection generally require good carrier mobility, high X-ray absorption rate, and low-cost preparation methods; the commercial X-ray detector materials are mainly silicon, cadmium, zinc telluride and a-Se materials. Detectors based on such materials are generally associated with the limitations of complicated synthesis and fabrication requirements, lower detection rate and sensitivity, etc. [1,3]. In recent years, organic–inorganic lead halide perovskites have been particularly favored by researchers for their excellent photoelectric properties, and have been widely used in solar cells [4,5,6], light-emitting diodes [7,8,9], and photodetectors [10,11,12,13]. At the same time, due to the strong absorption ability of lead halide perovskites on X-rays and the realization of low-cost solution preparation, it has become a hot spot in the field of X-ray detection. At present, there are two main obstacles affecting the practical application of lead halide perovskite devices: one is that the perovskite material contains toxic heavy metal lead; Pb leakage will bring great harm to the environment and human health, however, as an X-ray-absorbing material in the element selection, and require a large atomic number, such as Pb and other elements, so how to maintain the absorption of X-rays while reducing the harm to humans and the environment is an urgent problem to be solved. Second, organic–inorganic perovskites are extremely sensitive to light, temperature, and humidity, which will cause the decomposition of materials and seriously affect the stability of materials and devices. A large number of studies have also been carried out on these issues. With its excellent photoelectric properties and excellent thermal and moisture stability, all-inorganic perovskites have attracted more and more attention from researchers. Zeng et al. obtained a highly sensitive all-inorganic perovskite nanocrystalline X-ray detector by the solution method, and achieved a high sensitivity at a certain dose rate [14]. The low-cost traditional solution method has become one of the main processes for the preparation of all-inorganic perovskite, but the CsPbBr_3_ nanocrystals prepared by this method have the disadvantages of poor continuity, large defect density, and high concentration of the impurity phase, so researchers changed the crystallization performance of perovskites by doping CsPbBr_3_ so as to achieve the purpose of regulating morphology and improving the photoelectric performance. For ABX_3_-type inorganic perovskites, B-position doping usually has a stronger effect on photoelectric properties than A-position doping [15,16], and numerous studies have shown that metal doping can effectively improve crystallization properties and the carrier transport of CsPbX_3_ to improve the efficiency and stability of the device. Yang et al. [17] prepared light-emitting diodes by Cu^2+^ doping of CsPb(Br/Cl)_3_; the photoluminescence quantum yield was promoted to 94%, and the stability maintained for 30 days. Liu et al. introduced Li^+^ ions into the CsPbIBr_2_ lattice to obtain highly crystalline and well-oriented CsPbIBr_2_ crystals, and the photoelectric conversion efficiency of the doped CsPbIBr_2_ solar cells reached 9.25%, which was much higher than that of undoped devices (7.41%) [18]. Tang et al. introduced Ln^3+^ ions into the perovskite lattice; the grain size increased and the carrier lifetime was extended, which significantly improved the performance of inorganic CsPbBr_3_ solar cell devices, and the photoelectric conversion efficiency of 10.14% and the high open-circuit voltage of 1.594 V could be obtained under illumination. At the same time, it maintains good stability in the air [19]. Although some progress has been made in B-doped inorganic perovskites, most of the doped ions radii selected by these studies are quite different from the Pb^2+^ ion radius, which may bring about a sharp expansion or contraction of the crystal lattice, and then have a certain negative impact on the crystallization properties of the material. Theoretically, the radius of Sr^2+^ (132 pm) is close to that of Pb^2+^ (133 pm) [20,21], and the partial replacement of Pb^2+^ in CsPbBr_3_ by Sr^2+^ can maintain the unique crystal structure of perovskite and excellent photoelectric performance; at the same time, this reduces the amount of Pb^2+^ to achieve the goal of environmental protection. In our previous research work, we realized the fast detection of X-ray with less Pb CsPbBr_3_ thick film devices replaced by Sr^2+^ under a certain external bias (2 V, 5 V, 8 V) [22]. To achieve the sustainable requirement of low energy consumption of detectors, it is truly necessary to study self-powered detectors without external bias.

It has been demonstrated that a thick film of CsPbBr_3_ can make for an effective self-powered X-ray detector without the need for an external bias [23]; however, the previous study involved a heating step in the precursor solution preparation, which used up a fair amount of energy to add the preparation cost. For this reason, resolving to produce CsPbBr_3_ self-powered detectors at room temperature is particularly advantageous. In this work, base on a heteroatomic Sr^2+^ doping strategy, we prepared the all-inorganic perovskite thick films (CsPbBr_3_ and CsPbBr_3_:Sr) by an anti-solvent deposition method; the partial substitution of Pb^2+^ by Sr^2+^ increased the density of the perovskite thick film, which played the role of passivation defects, and the increase of chemical binding energy between elements also indicated a more stable phase structure with Sr^2+^. In addition, the prepared CsPbBr_3_:Sr X-ray detector showed high sensitivity (517.02 μC Gyair^−1^ cm^−3^), and steady current signals at different X-ray dose rate. This work provides a feasible and effective heteroatom substitution strategy for the preparation of CsPbBr_3_ X-ray detectors, and enhances the competitiveness of inorganic less-lead perovskite detectors in the field of photoelectric detection in the future. 

## 2. Materials and Methods

### 2.1. Materials

The 1 cm^2^ ITO substrates were produced by Advanced Election Technology Co., Ltd. The PbBr_2_, CsBr, SrBr_2_, poly(3,4-ethylenedioxythiophene)-poly(styrenesulfonate) (PEDOT:PSS) and [6,6]-phenyl-C61-butyric acid methyl ester (PCBM) were purchased from Aladdin-Holdings Group. Dimethyl formamide (DMF), dimethyl sulfoxide (DMSO), xylene, O-dichlorobenzene (ODB), and ethyl alcohol with the purity of 99% were purchased from Chengdu Cologne Chemicals Co., Ltd. (Chengdu, China).

### 2.2. Device Fabrication

**Figure 1:**
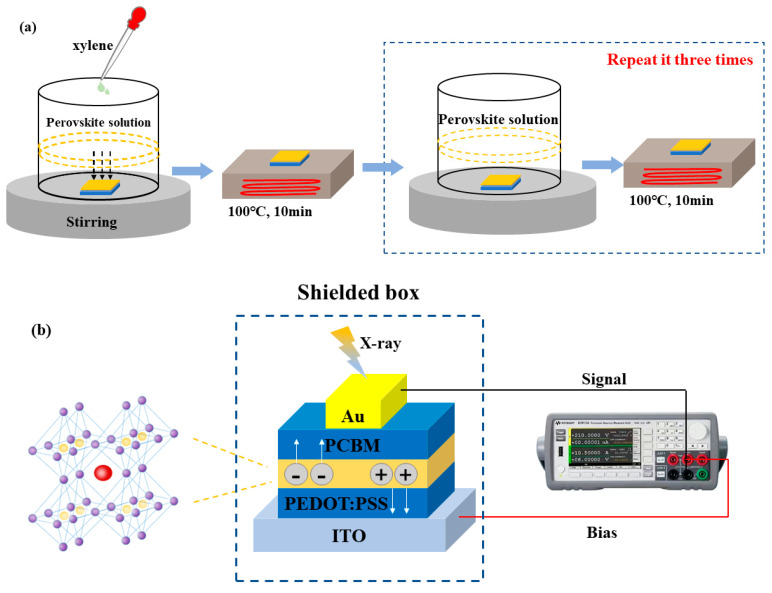
(**a**) Schematic of the perovskite thick film preparation and (**b**) photoelectric measurement under X-ray exposure.

ITO was cleaned with deionized water and ethanol for 20 min, respectively, and treated with ultraviolet ozone for 30 min. Then, PEDOT: PSS was spin-coated at 2000 rpm for 30 s as the hole transport layer, and annealed immediately at 130 °C for 10 min. All-inorganic CsPbBr_3_ and CsPbBr_3_:Sr were synthesized through an anti-solvent method described as the following steps: as shown in Figure 1, we dissolved PbBr_2_ and CsBr with a molar ratio of 1:1 in 10 mL of mixed solution of DMF and DMSO (7:3 by volume), to form 0.3 M perovskite precursor solution. The CsPbBr_3_:Sr precursors were prepared by 6 mol% SrBr_2_ substituting the same molar ratio of PbBr_2_. After stirring the solution at room temperature for 4 h, it was filtered throμGh 0.22 µm polytetrafluoroethylene filter membrane. The filtrate and ITO coated with a hole transport layer were then transferred to a glass container, and 5 mL of anti-solvent xylene was added dropwise to the container while stirred at 400 rpm min^−1^, after 20 min deposition, the substrate needed to be taken out vertically and immediately annealed at 100 °C for 10 min. In order to repair the perovskite surface, we transferred CsPbBr_3_ and CsPbBr_3_:Sr to perovskite solution and let it stand for 5 min after the substrate was cooled, and then the repaired perovskite was annealed at 100 °C for 10 min; this procedure was repeated three times. Then the CsPbBr_3_ and CsPbBr_3_:Sr thick films were prepared. The thickness of the films was controlled at 160 μm (estimated by cross-sectional images in Supporting Information, Figure S1). ODB solution with 2 wt% PCBM was then spun on the thick film surface as the electron transport layer, and annealed at 70 °C for 10 min. Finally, Au was sputtered onto this surface to form electrodes with an area of 1 mm^2^ to produce the device. 

### 2.3. Characterizations

The crystal structure and phase composition were determined using an DX2700 instrument X-ray diffractometer (XRD)with Cu Kα radiation (λ = 1.5406 Å). The surface morphology and elemental composition of the thick films were tested by observing Scanning Electron Microscope (SEM) images via the instrument of Phenom-World (Phenom XL, The Netherlands). The binding energy of the thick films was analyzed by X-ray photoelectron spectroscopy (XPS), which was tested by a Thermo Scientific NEXSA instrument (ThermoFisher, US). The photoluminescence (PL) spectra of the thick films were measured on a SHIMADZU RF2501 instrument, (shimadzu, Japan). Eventually, an Agilent B2912A semiconductor parameter analyzer(Agilent, US) was used to analyze the photoresponse characteristics of the devices under X-ray excitation. 

## 3. Results and Discussion

**Figure 2:**
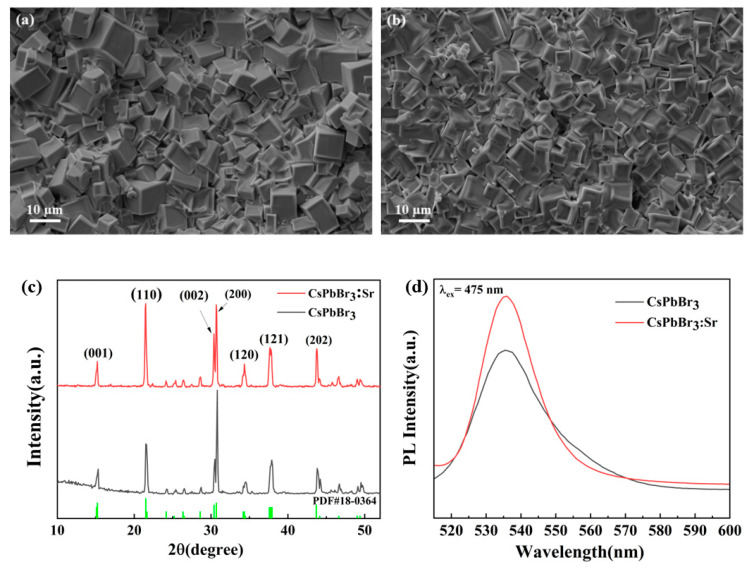
The SEM images of CsPbBr_3_ (**a**) and CsPbBr_3_:Sr (**b**), (**c**) XRD patterns, and (**d**) PL spectra of perovskite thick films.

Figure 2a,b show the SEM images of CsPbBr_3_ and CsPbBr_3_:Sr films, respectively. It is not difficult to find that the original CsPbBr_3_ film was composed of a large number of cubes closely stacked, with different particle sizes (about 5–10 μm), and also contained a small number of pores due to solvent evaporation. After Sr substitution, the grains retained the original cubic phase and the particle size decreased (about 5 μm). On the surface of the film, the edges and corners of the cube began to melt and were closely linked with the neighboring grains. From the SEM images, it can be clearly shows that the more uniform, compact, and flat films were displayed in CsPbBr_3_:Sr. This indicates that the partial substitution of Sr^2+^ for Pb^2+^ greatly increases the density of thick films and decreases the number of grain boundaries; for perovskite materials, the existence of a large number of grain boundaries became the center of electron-hole recombination and the channel of leakage current [23], which is not conducive to charge transfer, and the increase of dark current affected the practical use of the detector. Therefore, it can be concluded that the film-forming quality of perovskite thick film is greatly improved after partial substitution of Sr.

The X-ray diffraction (XRD) pattern of the CsPbBr_3_, and CsPbBr_3_:Sr showed seven main sharp diffraction peaks at 15.2°, 25.6°, 30.4°, 30.7°, 34.3°, 37.7°, and 43.7°, as shown in Figure 2c, which can be assigned to the (001), (110), (002), (200), (120), (121), and (202) planes, respectively [24]. The XRD results corresponded well with the results of cubic phase (PDF#18-0364). No additional diffraction was observed in CsPbBr_3_:Sr, indicating no new crystalline phase was formed. With the addition of Sr, the intensity of the characteristic diffraction peak of CsPbBr_3_ decreased slightly, which indicates that the substitution of Sr inhibited the grains’ growth to a certain extent; this result is consistent with the XRD results. Meanwhile, the preferential orientation of the growth of perovskite grain showed an obvious change; the grain growth from the original along (200) crystal plane to (110) crystal plane changed, and the grain growth in the vertical direction ensured the vertical migration of the photo-generated carriers [25,26]. The charge extraction of the two-terminal transport layer was promoted, which is beneficial to the improvement of the device performance. Photoluminescence spectroscopy is one of the most effective means to investigate defects of semiconductors. Figure 2d shows the photoluminescence spectrum of perovskite thick films. Under the excitation of 475 nm light source, all samples have obvious luminescence peak at 535 nm, compared with unsubstituted CsPbBr_3_ thick film, the fluorescence emission intensity of CsPbBr_3_:Sr was stronger, which indicates that there was less non-radiative recombination inside the CsPbBr_3_:Sr structure, predicting a lower defect state density [27,28] and suggesting the Sr substitution has a certain passivation effect on the defects in CsPbBr_3_.

**Figure 3:**
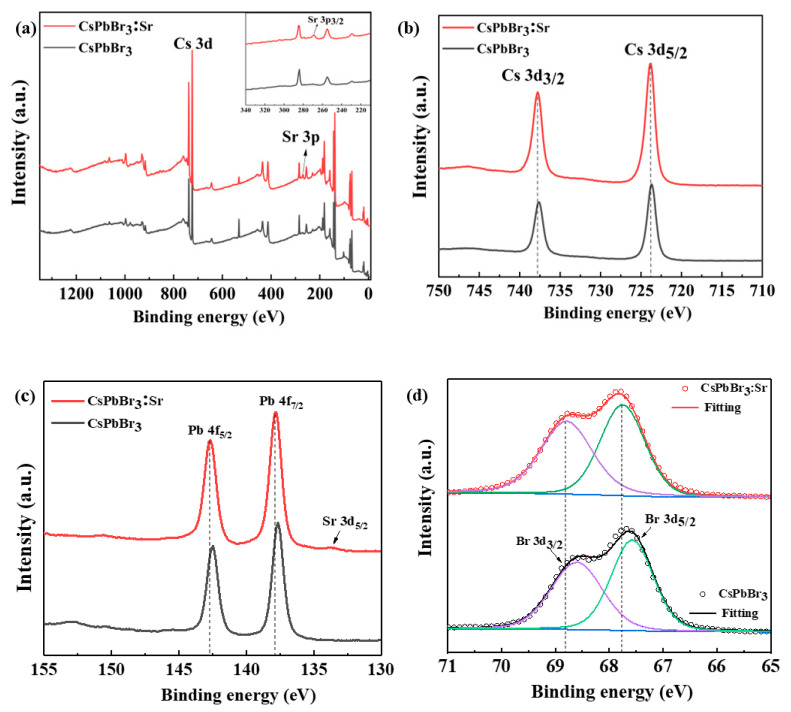
The XPS of CsPbBr_3_ and CsPbBr_3_:Sr thick films (**a**), Cs 3d (**b**), Pb 4f (**c**), and Br 3d (**d**).

XPS is used to study the state of the elements and the binding energy. From the full spectrum of XPS (Figure 3a), it can be seen that the main components of CsPbBr_3_ and CsPbBr_3_:Sr thick films were similar; Figure 3b–d shows the XPS profiles of Cs 3d, Pb 4f, and Br 3d in the thick films. Interestingly, a characteristic peak of Sr^2+^ 3p_3_ at the binding energy of 269.0 eV in CsPbBr_3_:Sr, as illustrated in Figure 3a, and Figure 3c showed two strong characteristic peaks around 138 eV, 142 eV for both samples, corresponding to the characteristic signals of Pb 4f_7/2_ and Pb 4f_5/2_, while a characteristic peak of Sr 3d_5_ appeared at the binding energy of 133.7 eV, thus confirming the existence of Sr^2+^ [29]. From the XPS spectra, it can be seen that the strength of characteristic peaks of all elements increased and moves towards higher binding energy. The characteristic peaks corresponding to each element (Cs, Pb, Br and Sr) of CsPbBr_3_ and CsPbBr_3_:Sr thick films are recorded in Table S1. The results showed that the binding energy of each element in CsPbBr_3_ is increased after Sr substitution, which indicates that the chemical binding energies between Cs, Pb, and Br atoms was enhanced, which is beneficial to improve the phase stability of CsPbBr_3_ [30].

**Figure 4:**
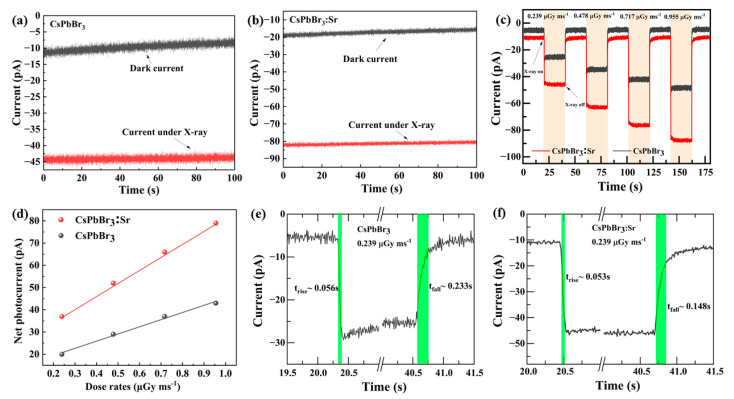
(**a**,**b**) IT characteristics of the devices with and without X-ray exposure, (**c**) the response to X-ray on turning the X-ray source on and off under zero bias, (**d**) the net current at different dose rates, (**e**,**f**) the response speed of CsPbBr_3_ and CsPbBr_3_:Sr devices.

CsPbBr_3_ and CsPbBr_3_:Sr were exposed to an X-ray source, and the distance between the X-ray window and the device was fixed at 10 cm to test the electrical properties of the detectors, including dark current, response to X-ray and other parameters. Figure S2 shows the I-V curves of the device in the dark and light with the external bias from −1 V to 1 V. The I-V curve that does not cross zero point indicating that the device can operate without an additional energy [31]. Under the X-ray illumination (60 kV, tube current 0.3 mA), the I-T curves (Figure 4a,b) of the device show the light and dark current variations over 100 s. The dark current of CsPbBr_3_ was about 10 pA, the light current was about 45 pA, and the net light current was about 35 pA by subtracting the dark current from the light current, and under the same conditions, the dark current of CsPbBr_3_:Sr was about 20 pA, and the light current was 83 pA. The I-T curve showed that the device displayed a good stability of the current signal in a limited period of time at a fixed X-ray tube voltage and tube current. 

The attenuation efficiency and thickness curve toward 35 keV X-rays for several typical semiconductors is depicted in Figure S3. It was found that the attenuation coefficient of CsPbBr_3_ was higher than MAPbBr_3_, a-Se, and Si. The film thickness of 160 µm was enough to attenuate 74.5% of 35 keV X-rays. To further test the time-dependent response of devices to different doses of X-rays, we fixed X-ray tube voltages of 60 kV and tube currents of 0.1 mA, 0.2 mA, 0.3 mA, and 0.4 mA, corresponding to 0.239, 0.478, 0.717, 0.955 µGy ms^−1^, respectively, as shown in Figure 4c. It is easy to see that the CsPbBr_3_ and CsPbBr_3_:Sr detectors showed better photoelectric responses at a bias of 0 V under different X-ray dose rates. The dark current of the CsPbBr_3_ detector was about 5 pA, and the dark current of the CsPbBr_3_:Sr detector was about 10 pA; because the Sr^2+^ substitution in the perovskite increases the conductivity of the material to some extent, when the X-ray dose rate was increased from 0.239 μGy ms^−1^ to 0.955 µGy ms^−1^, both detectors maintained a lower dark current and better on and off properties. Because of the repair of the grain boundaries of CsPbBr_3_ thick films after Sr^2+^ substitution, the defects of electron-hole trapping were reduced, the charge transfer to the two transport layers was easier, and the response of CsPbBr_3_:Sr detector at different X-ray dose was obviously enhanced. To the CsPbBr_3_:Sr detector, the ratio of photocurrent and dark current ($I_{photo}/I_{dark}$) increased from original CsPbBr_3_ (3–5) to (4.3–8.5). 

The curve of the net photocurrent is shown in Figure 4d; both CsPbBr_3_ and CsPbBr_3_:Sr detectors showed a good linear relationship with different X-ray dose rates. The net photocurrent of CsPbBr_3_:Sr had a large enhancement, and the photoresponse signal was enhanced by 75–79%, while the X-ray dose rate increased from 0.239 μGy ms^−1^ to 0.955 μGy ms^−1^. For the detector, the response time is an important parameter to reflect its response to the incident X-ray, which is closely related to the effective carrier extraction and recombination; T_rise_ and T_fall_ were defined as the time it takes for the photocurrent to rise from 10% to 90% and to fall from 90% to 10%. As shown in Figure 4e,f, the response curve of the device was extracted when the X-ray dose was 0.239 μGy ms^−1^. The T_rise_ and T_fall_ of the CsPbBr_3_ detectors were 0.056 s and 0.233 s, respectively. The response speed of CsPbBr_3_:Sr detectors was obviously promoted, and their T_rise_ and T_fall_ were shortened to 0.053 s and 0.148 s, respectively. Therefore, the partial substitution of Sr^2+^ for Pb^2+^ improved the carrier extraction efficiency and response speed of the detectors greatly.

The sensitivity is one of the most important parameters of the detectors, which is calculated by the formula S = Q/(A·X), where *Q* is the charge received in the course of radiation, *A* is the dose of X-ray radiation, and *X* is the volume of the radiation-receiving region. As can be seen from Figure S4, the sensitivity of the CsPbBr_3_ was 288.11 μC Gy_air_^−1^ cm^−3^ at zero bias, and it is clear that the CsPbBr_3_:Sr detector had higher sensitivity, being able to reach 517.02 μC Gy_air_^−1^ cm^−3^; although this value is small, we would like to point out that X-ray detectors are selected not only based on their sensitivity, but also according to other more important parameters such as SNR and dark current. SNR is the ratio of device signal current-to-noise current *SNR* = I*_signal_*/I*_noise_*, where I*_signal_* can be obtained by subtracting the average dark current from the average photocurrent, I*_noise_* can be obtained from calculating the standard deviation of the light current, and the calculation formula is as follows: $I_{noise}=\sqrt{1/N\sum_{i}^{N}\left(I_{i}-{\overset{-}{I}}_{photo}\right)}$. The properties of X-ray detectors are summarized in Table 1. It is easy to see that SNR of CsPbBr_3_ and CsPbBr_3_:Sr detectors were much higher than previously reported [23] and the detectors in this work had a low dark current and a high signal-to-noise ratio compared with other devices. In addition, comparing CsPbBr_3_ with CsPbBr_3_:Sr detectors, the latter exhibited a fast response time with a high sensitivity at zero bias. In a word, the CsPbBr_3_:Sr and its device are potential candidates for the development of self-powered inorganic perovskite X-ray detectors.

## 4. Conclusions

In summary, a heteroatomic Sr^2+^ doping strategy achieved compact and homogeneous CsPbBr_3_ thick films. The Sr^2+^ doping was obviously beneficial for passivating the defects produced by a large number of grain boundaries in perovskite, and the ordered growth along the vertical direction was advantageous to the charge transfer and extraction. Further, the self powered CsPbBr_3_:Sr detector exhibited a higher ratio of photocurrent and dark current, with a sensitivity of 517.02 μC Gy_air_^−1^ cm^−3^, while maintaining a faster response (0.053–0.148 s). These excellent characteristics indicate that the substitution of Pb^2+^ by alkali metal Sr^2+^ has great potential for use in Pb-less self-powered X-ray detectors with.

## Figures and Tables

**Table 1:** Comparison of the relevant device parameters of the X-ray detectors.

Material	Bias (V)	SC/Film	Absorbing Thickness	Dark Current	The Highest Detectable Dose Rate	Sensitivity (µC Gy_air_^−1^ cm^−3^ )	SNR	Response Time	Ref
MAPbBr_3_	0.1	SC	2–3 mm	29 nA cm^−2^	/	270	/	216 µs	[32]
MAPbI_3_	200	PC	1 mm	6 µA cm^−1^	/	25,300	/	/	[33]
CsPbBr_3_	0	film	80 µm	/	0.082 μGy ms^−1^	/	39.2	/	[23]
MAPbI_3_	80	film	60 µm	/	/	25	/	/	[34]
Cs_2_AgBiBr_6_	100	SC	2 mm	/	61.12 μGy S^−1^	1600	/	/	[35]
Sb_2_Se_3_	−1	film	5 µm	91.2 nA	5.499 µGy S^−1^	21,000	/	2.5 ms	[36]
Cs_3_Bi_2_Br_3_I_6_	50	PC	0.7 mm	12.5 nA	2.2 mGy s^−1^	8.16	/	/	[37]
BiI_2_	10	film	4–8 μm	20 pA	/	/	/	231–368 ms	[38]
CsPbBr_3_	0	film	160 µm	5 pA	0.955 μGy ms^−1^	288.11	58.8	0.056–0.233 s	this work
CsPbBr_3_:Sr	0	film	160 µm	12 pA	0.955 μGy ms^−1^	517.02	154.9	0.053–0.148 s	this work

Notes. SC: single crystal, PC: polycrystalline.

## Data Availability

The data presented in this study are available on request from the corresponding author.

## Supplementary Materials

The following supporting information can be downloaded at: https://www.mdpi.com/article/10.3390/ma16051783/s1, Figure S1: The cross-sectional images of CsPbBr_3_ (a) and CsPbBr_3_:Sr (b); Figure S2: Current-voltage curves of the CsPbBr_3_ (a) and CsPbBr_3_:Sr devices (b); Figure S3: Attenuation efficiency of different materials for the X-ray photon energy of 60 keV; Figure S4: The sensitivity of the CsPbBr_3_ and CsPbBr_3_:Sr devices under different dose rate at zero bias; Table S1: XPS Characteristic peak positions corresponding to each element (eV).

## References

[1] Kasap S., Frey J.B., Belev G., Tousignant O., Mani H., Greenspan J., Laperriere L., Bubon O., Reznik A., DeCrescenzo G. (2011). Amorphous and Polycrystalline Photoconductors for Direct Conversion Flat Panel X-ray Image Sensors. Sensors.

[2] Yaffe M.J., Rowlands J.A. (1997). X-ray detectors for digital radiography. Phys. Med. Biol..

[3] Kasap S.O., Haugen C., Nesdoly M., Rowlands J.A. (2000). Properties of a-Se for use in at panel X-ray image detectors. J. Non-Cryst. Solids.

[4] Chen H., Teale S., Chen B., Hou Y., Grater L., Zhu T., Bertens K., Park S.M., Atapattu H.R., Gao Y. (2022). Quantum-size-tuned heterostructures enable efficient and stable inverted perovskite solar cells. Nat. Photonics.

[5] Hossain M.I., Shahiduzzaman M., Saleque A.M., Huqe R., Qarony W., Ahmed S., Akhtaruzzaman M., Knipp D., Tsang Y.H., Taima T. (2021). Improved Nanophotonic Front Contact Design for High-Performance Perovskite Single-Junction and Perovskite/Perovskite Tandem Solar Cells. Sol. RRL.

[6] Yuce H., Perini C.A., Hidalgo J., Castro-Méndez A.F., Evans C., Betancur P.F., Vagott J.N., An Y., Bairley K., Demir M.M. (2022). Understanding the impact of SrI2 additive on the properties of Sn-based halide perovskites. Opt. Mater..

[7] Shen L., Fang Y., Wang D., Bai Y., Deng Y., Wang M., Lu Y., Huang J. (2016). A Self-Powered, Sub-nanosecond-Response Solution Processed Hybrid Perovskite Photodetector for Time Resolved Photoluminescence-Lifetime Detection. Adv. Mater..

[8] Shi Y., Wu W., Dong H., Li G., Xi K., Divitini G., Ran C., Yuan F., Zhang M., Jiao B. (2018). A Strategy for Architecture Design of Crystalline Perovskite Light-Emitting Diodes with High Performance. Adv. Mater..

[9] Thapa S., Adhikari G.C., Zhu H., Grigoriev A., Zhu P. (2019). Zn-Alloyed All-Inorganic Halide Perovskite-Based White Light Emitting Diodes with Superior Color Quality. Sci. Rep..

[10] Dou L., Yang Y., You J., Hong Z., Chang W.-H., Li G. (2014). Solution-processed hybrid perovskite photodetectors with high detectivity. Nat. Commun..

[11] He Y., Hadar I., De Siena M.C., Klepov V.V., Pan L., Chung D.Y., Kanatzidis M.G. (2022). Sensitivity and Detection Limit of Spectroscopic-Grade Perovskite CsPbBr_3_ Crystal for Hard X-ray Detection. Adv. Energy Mater..

[12] Zhou L., Lu X., Wu J., Jiang H., Chen L., Ouyang X., Lau K.M. (2018). Self-Powered Fast-Response X-ray Detectors Based on Vertical GaN p-n Diodes. IEEE Electron. Device Lett..

[13] Heo J.H., Park J.K., Yang Y.M., Lee D.S., Im S.H. (2021). Self-powered flexible all-perovskite X-ray detectors with high sensitivity and fast response. ISCIENCE.

[14] Li X., Meng C., Huang B., Yang D., Xu X., Zeng H. (2020). All-Perovskite Integrated X-ray Detector with Ultrahigh Sensitivity. Adv. Opt. Mater..

[15] Jin J., Li H., Chen C., Zhang B., Xu L., Dong B., Song H., Dai Q. (2017). Enhanced Performance of Perovskite Solar Cells with Zinc Chloride Additives. ACS Appl. Mater. Interfaces.

[16] Xu L., Yuan S., Zeng H., Song J. (2019). A comprehensive review of doping in perovskite nanocrystals/quantum dots: Evolution of structure, electronics, optics, and light-emitting diodes. Mater. Today Nano.

[17] Gao Y., Luo C., Yan C., Li W., Liu C., Yang W. (2022). Copper-doping defect-lowered perovskite nanosheets for deep-blue light-emitting diodes. J. Colloid Interface Sci..

[18] Tan X., Liu X., Liu Z., Sun B., Li J., Xi S., Shi T., Tang Z., Liao G. (2020). Enhancing the optical, morphological and electronic properties of the solution-processed CsPbIBr_2_ films by Li doping for efficient carbon-based perovskite solar cells. Appl. Surf. Sci..

[19] Duan J., Zhao Y., Yang X., Wang Y., He B., Tang Q. (2018). Lanthanide Ions Doped CsPbBr_3_ Halides for HTM-Free 10.14%-Efficiency Inorganic Perovskite Solar Cell with an Ultrahigh Open-Circuit Voltage of 1.594 V. Adv. Energy Mater..

[20] Jacobsson T.J., Pazoki M., Hagfeldt A., Edvinsson T. (2015). Goldschmidt’s Rules and Strontium Replacement in Lead Halogen Perovskite Solar Cells: Theory and Preliminary Experiments on CH_3_NH_3_SrI_3_. J. Phys. Chem. C.

[21] Chan S.H., Wu M.C., Lee K.M., Chen W.C., Lin T.H., Su W.F. (2017). Enhancing perovskite solar cell performance and stability by doping barium in methylammonium lead halide. J. Mater. Chem. A.

[22] Liu C., Yang D., Tian H., Zhang W., Zhu J. (2023). X-ray detectors based on CsPb_1-x_Sr_x_Br_3_ thick films. Ceram. Int..

[23] Gou Z., Huanglong S., Ke W., Sun H., Tian H., Gao X., Zhu X., Yang D., Wangyang P. (2019). Self-Powered X-ray Detector Based on All-Inorganic Perovskite Thick Film with High Sensitivity Under Low Dose Rate. Phys. Status Solidi (RRL) Rapid Res. Lett..

[24] Castro-Méndez A.F., Hidalgo J., Correa-Baena J.P. (2019). The Role of Grain Boundaries in Perovskite Solar Cells. Adv. Energy Mater..

[25] Zhu W., Zhang Q., Chen D., Zhang Z., Lin Z., Chang J., Zhang J., Zhang C., Hao Y. (2018). Intermolecular Exchange Boosts Efficiency of Air-Stable, Carbon-Based All-Inorganic Planar CsPbIBr_2_ Perovskite Solar Cells to Over 9%. Adv. Energy Mater..

[26] Liu C., Li W., Zhang C., Ma Y., Fan J., Mai Y. (2018). All-Inorganic CsPbI_2_Br Perovskite Solar Cells with High Efficiency Exceeding 13%. J. Am. Chem. Soc..

[27] Gao L., Huang S., Chen L., Li X., Ding B., Huang S., Yang G. (2018). Excellent Stability of Perovskite Solar Cells by Passivation Engineering. Solar RRL.

[28] Wu J., Xu X., Zhao Y., Shi J., Xu Y., Luo Y., Li D., Wu H., Meng Q. (2017). DMF as an Additive in a Two-Step Spin-Coating Method for 20% Conversion Efficiency in Perovskite Solar Cells. ACS Appl. Mater. Interfaces.

[29] Shai X., Zuo L., Sun P., Liao P., Huang W., Yao E.-P., Li H., Liu S., Shen Y., Yang Y. (2017). Efficient planar perovskite solar cells using halide Sr-substituted Pb perovskite. Nano Energy.

[30] Nam J.K., Chai S.U., Cha W., Choi Y.J., Kim W., Jung M.S., Kwon J., Kim D., Park J.H. (2017). Potassium Incorporation for Enhanced Performance and Stability of Fully Inorganic Cesium Lead Halide Perovskite Solar Cells. Nano Lett..

[31] Yan J., Gao F., Tian Y., Li Y., Gong W., Wang S., Zhu H., Li L. (2022). Controllable Perovskite Single Crystal Heterojunction for Stable Self-Powered Photo-Imaging and X-ray Detection. Adv. Opt. Mater..

[32] Wei H., Fang Y., Mulligan P., Chuirazzi W., Fang H.-H., Wang C., Ecker B.R., Gao Y., Loi M.A., Cao P.M.W.C.L. (2016). Sensitive X-ray detectors made of methylammonium lead tribromide perovskite single crystals. Nat. Photonics.

[33] Shrestha S., Fischer R., Matt G.J., Feldner P., Michel T., Osvet A., Levchuk I., Merle B., Golkar S., Chen H. (2017). High-performance direct conversion X-ray detectors based on sintered hybrid lead triiodide perovskite wafers. Nat. Photonics.

[34] Yakunin S., Sytnyk M., Kriegner D., Shrestha S., Richter M., Matt G.J., Azimi H., Brabec C.J., Stangl J., Kovalenko M.V. (2015). Detection of X-ray photons by solution-processed organic-inorganic perovskites. Nat. Photonics.

[35] Steele J.A., Pan W., Martin C., Keshavarz M., Debroye E., Yuan H., Banerjee S., Fron E., Jonckheere D., Kim C.W. (2018). Photophysical Pathways in Highly Sensitive Cs_2_AgBiBr_6_Double-Perovskite Single-Crystal X-ray Detectors. Adv. Mater..

[36] Wang C., Du X., Wang S., Deng H., Chen C., Niu G., Pang J., Li K., Lu S., Lin X. (2021). Sb_2_Se_3_ film with grain size over 10 microm toward X-ray detection. Front. Optoelectron..

[37] Daum M., Deumel S., Sytnyk M., Afify H.A., Hock R., Eigen A., Zhao B., Halik M., These A., Matt G.J. (2021). Self-Healing Cs_3_Bi_2_Br_3_I_6_ Perovskite Wafers for X-ray Detection. Adv. Funct. Mater..

[38] Sun H., Yang D., Liu Y., Zhu X. (2019). Highly Flexible X-ray Detectors Based on Pure Inorganic Metal Iodide Polycrystalline Thin Films as Photon-to-Charge Conversion Layers. ACS Appl. Electron. Mater..

