# Supplementary material for: Sr-Doping All-Inorganic CsPbBr3 Perovskite Thick Film for Self-Powered X-ray Detectors
Source: Materials (Basel). 2023 Feb 21;16(5):1783. doi: 10.3390/ma16051783 (PMC10003980; doi:10.3390/ma16051783)
Supplement: Supplementary file 1 [file materials-16-01783-s001.zip › materials-2193783-supplementary.pdf]

Supplementary Information

Sr-Doping All-Inorganic $CsPbBr_3$ Perovskite Thick Film for Self-Powered X-ray Detectors

Chuanqi Liu [1,2], Wen Zhang [2], Dingyu Yang [2], Haibo Tian [2] and Jun Zhu [1,*]

[1] College of Physics, Sichuan University, Chengdu 610065, China
[2] College of Optoelectronic Engineering, Chengdu University of Information Technology, Chengdu 610225, China
[*] Correspondence: authors: zhujun01@163.com

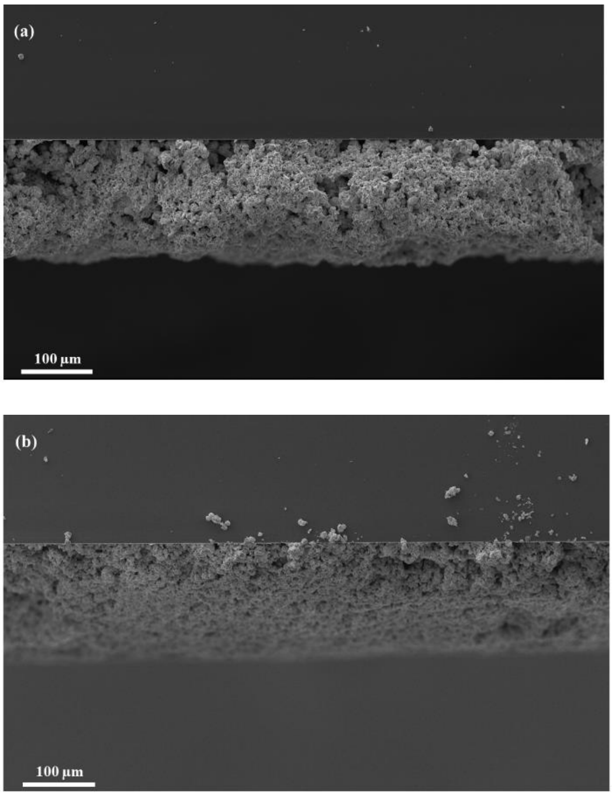

Figure S1. The cross-sectional images of $CsPbBr_3$ (**a**) and $CsPbBr_3$:Sr (**b**).

Table S1. XPS Characteristic peak positions corresponding to each element (eV).

	Sr $3p_3$	Sr $3d_5$	Cs $3d_{5/2}$	Cs $3d_{3/2}$	Pb $4f_{5/2}$	Pb $4f_{7/2}$	Br $3d_{5/2}$	Br $3d_{3/2}$
$CsPbBr_3$:Sr	268.3	133.75	723.86	737.78	142.69	137.84	67.81	68.78
$CsPbBr_3$	-	-	723.68	738.10	142.48	138.61	67.66	68.67

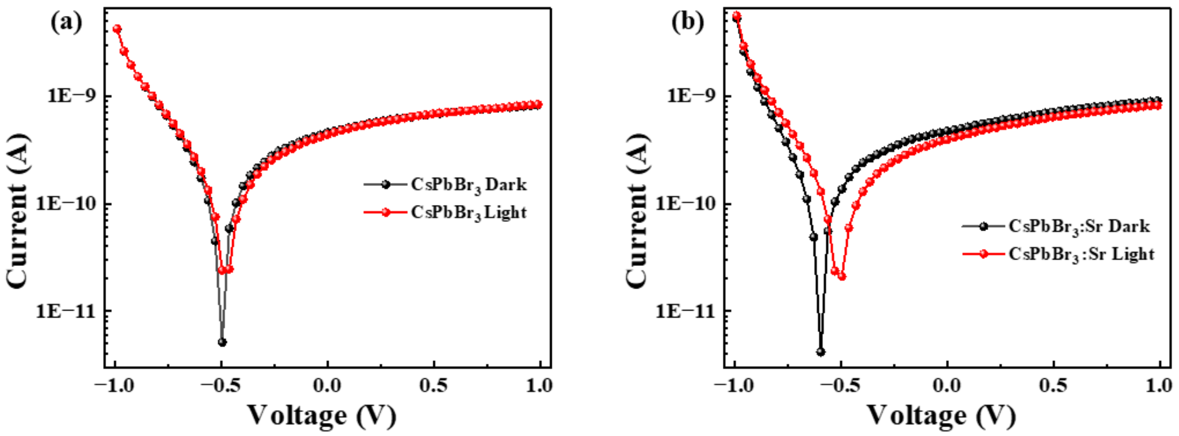

Figure S2. Current-voltage curves of the $CsPbBr_3$ (**a**) and $CsPbBr_3$:Sr devices (**b**).

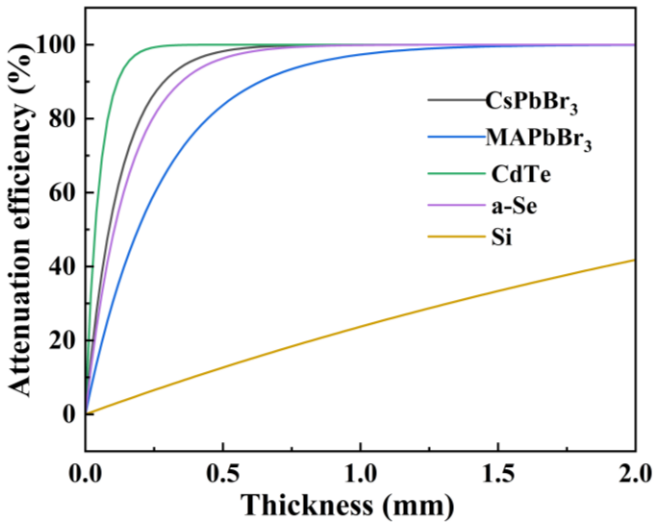

Figure S3. Attenuation efficiency of different materials for the X-ray photon energy of 60 keV.

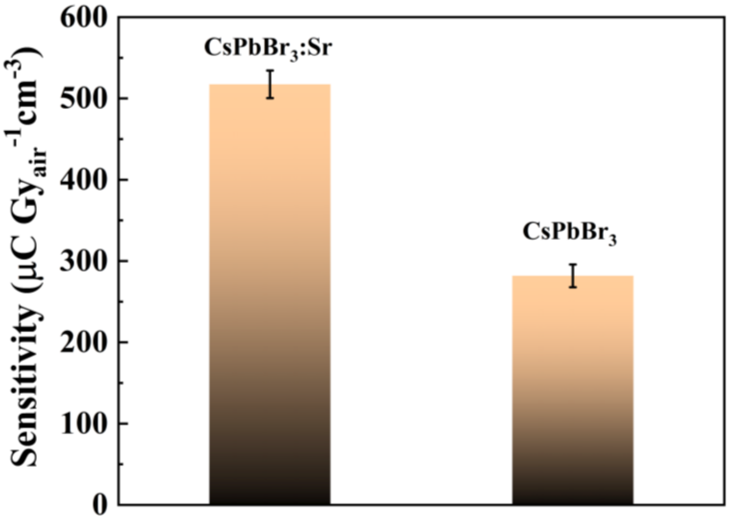

Figure S4. The sensitivity of the $CsPbBr_3$ and $CsPbBr_3$:Sr devices under different dose rate at zero bias.
